# Gene Dosage Effects at the Imprinted *Gnas* Cluster

**DOI:** 10.1371/journal.pone.0065639

**Published:** 2013-06-18

**Authors:** Simon T. Ball, Michelle L. Kelly, Joan E. Robson, Martin D. Turner, Jackie Harrison, Lynn Jones, Diane Napper, Colin V. Beechey, Tertius Hough, Antonius Plagge, Bruce M. Cattanach, Roger D. Cox, Jo Peters

**Affiliations:** 1 Medical Research Council Mammalian Genetics Unit, Harwell Science and Innovation Campus, Harwell, Oxfordshire, United Kingdom; 2 Medical Research Council Mary Lyon Centre, Harwell Science and Innovation Campus, Harwell, Oxfordshire, United Kingdom; 3 Department of Cellular and Molecular Physiology, Institute of Translational Medicine, University of Liverpool, Liverpool, United Kingdom; Universität des Saarlandes, Germany

## Abstract

Genomic imprinting results in parent-of-origin-dependent monoallelic gene expression. Early work showed that distal mouse chromosome 2 is imprinted, as maternal and paternal duplications of the region (with corresponding paternal and maternal deficiencies) give rise to different anomalous phenotypes with early postnatal lethalities. Newborns with maternal duplication (MatDp(dist2)) are long, thin and hypoactive whereas those with paternal duplication (PatDp(dist2)) are chunky, oedematous, and hyperactive. Here we focus on PatDp(dist2). Loss of expression of the maternally expressed *Gnas* transcript at the *Gnas* cluster has been thought to account for the PatDp(dist2) phenotype. But PatDp(dist2) also have two expressed doses of the paternally expressed *Gnasxl* transcript. Through the use of targeted mutations, we have generated PatDp(dist2) mice predicted to have 1 or 2 expressed doses of *Gnasxl*, and 0, 1 or 2 expressed doses of *Gnas*. We confirm that oedema is due to lack of expression of imprinted *Gnas* alone. We show that it is the combination of a double dose of *Gnasxl*, with no dose of imprinted *Gnas*, that gives rise to the characteristic hyperactive, chunky, oedematous, lethal PatDp(dist2) phenotype, which is also hypoglycaemic. However PatDp(dist2) mice in which the dosage of the *Gnasxl* and *Gnas* is balanced (either 2∶2 or 1∶1) are neither dysmorphic nor hyperactive, have normal glucose levels, and are fully viable. But PatDp(dist2) with biallelic expression of both *Gnasxl* and *Gnas* show a marked postnatal growth retardation. Our results show that most of the PatDp(dist2) phenotype is due to overexpression of *Gnasxl* combined with loss of expression of *Gnas*, and suggest that *Gnasxl* and *Gnas* may act antagonistically in a number of tissues and to cause a wide range of phenotypic effects. It can be concluded that monoallelic expression of both *Gnasxl* and *Gnas* is a requirement for normal postnatal growth and development.

## Introduction

Early work showed that certain chromosomal regions can lead to developmental abnormalities when both copies are exclusively maternally or paternally derived. Distal mouse chromosome 2 was one of the first such imprinting regions described, providing evidence that imprinting must affect expression of some genes according to parental origin [1]. Mice with two maternally derived copies of the region but no paternally derived ones, MatDp(dist2), had long thin bodies, failed to suckle, became inert and died within a few hours of birth. On the other hand, mice with two paternally derived copies of the region, but none that were maternally derived, PatDp(dist2), had an apparently opposite phenotype for they had short square bodies, (probably due to the combined effects of oedema and a chunky body shape), were notably hyperactive and died within a few days of birth. Hyperactivity may not be evident at birth but generally developed during the day of birth and became more pronounced in following days. In addition many PatDp(dist2) mice have an unusual front foot movement described as paddle feet, and a tail kink characteristically occurring about halfway down the tail [1], [2]. From genetic approaches using reciprocal translocations the region on mouse chromosome 2 giving rise to these imprinting effects was estimated to be 7 Mb in size and contained the *Gnas* cluster [3].

**Figure 1:**
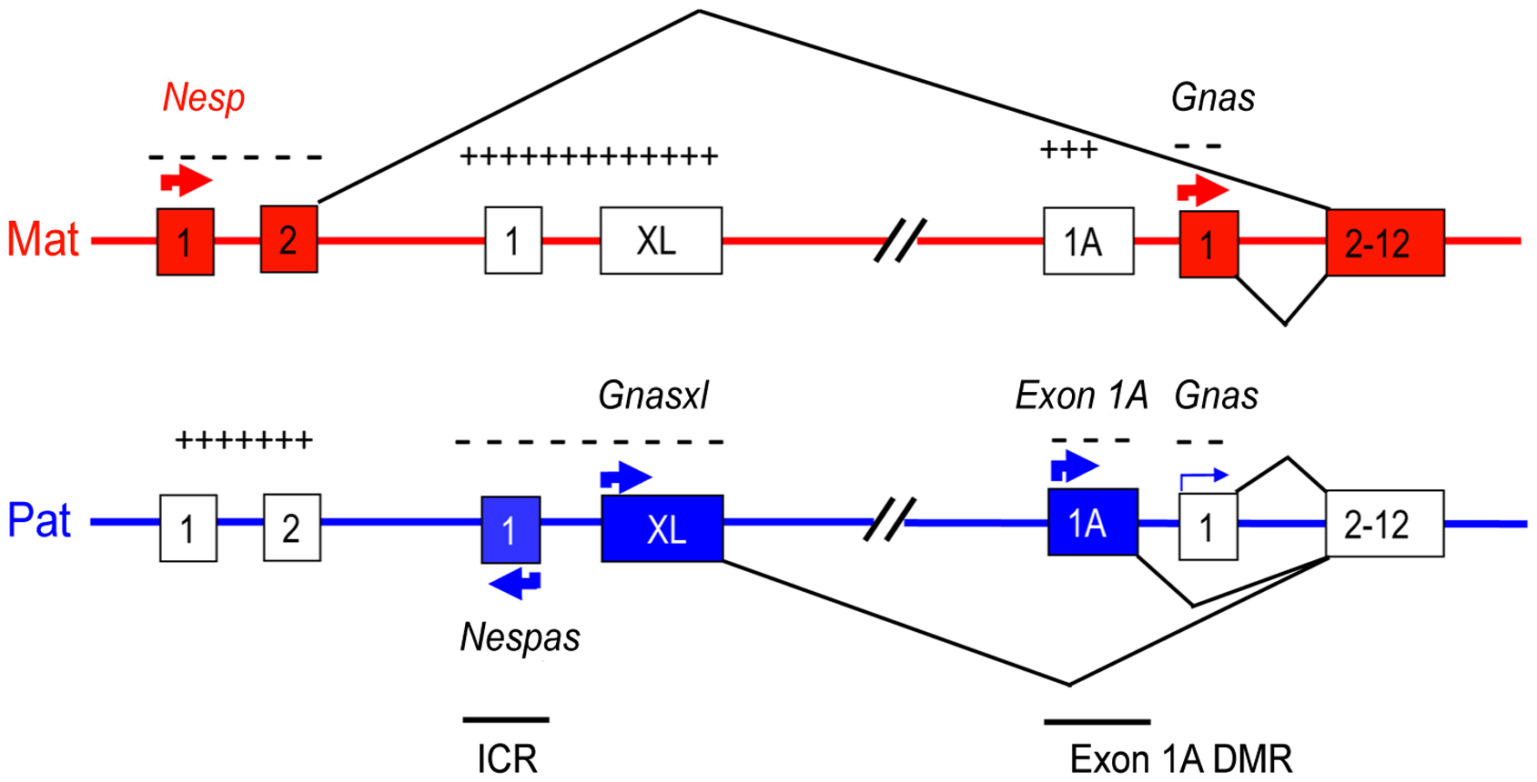
Summary of transcription and methylation at the *Gnas* cluster. Transcribed exons are shown as filled boxes and nontranscribed exons as empty boxes. *Nespas* and *Exon 1A* give rise to noncoding transcripts. The arrows indicate the direction of transcription and a thin arrow indicates low expression. The methylation status of the *Exon 1A* differentially methylated region (DMR) and the *Nespas* DMR (the imprinting control region, ICR, for the cluster) is shown as plus signs for methylated and minus signs for unmethylated.

Misexpression of transcripts at the imprinted *Gnas* cluster can account for much of the phenotype in both MatDp(dist2) and PatDp(dist2). *Gnas* is a complex imprinted gene cluster with three promoter regions that give rise to protein coding transcripts Nesp, Gnasxl and Gnas. Each of these transcripts has a unique first exon that splices on to a common set of downstream exons (Figure 1 **)**. *Nesp* is exclusively expressed from the maternal allele and *Gnasxl* from the paternal allele. *Gnas* is biallelically expressed in most tissues but is preferentially maternally expressed in some [4], [5], [6], [7]. *Nesp* gives rise to NESP55, neuroendocrine secretory protein 55, *Gnas* to Gsα, the alpha subunit of the Gs signalling protein and *Gnasxl* to XLαs, a variant form of Gsα which can also function as the alpha subunit of the heterotrimeric Gs protein. Both XLαs and Gsα can act on adenylyl cyclase to induce cyclic AMP production [8]. Although XLαs and Gsα share biochemical properties there is evidence that they act antagonistically and have opposite effects on metabolic regulation [9], [10], [11]. Overall the effects of paternally expressed *Gnasxl* in offspring result in increased demands on the mother (growth promoting) whereas the effects of maternally expressed *Gnas* result in fewer demands, in accordance with the conflict hypothesis [12].

Imprinted expression of protein coding transcripts at the *Gnas* cluster is controlled by an imprinting control region (ICR), a germline differentially methylated region called the *Nespas* DMR that covers the promoter of a noncoding antisense transcript, Nespas [13]. The *Nespas* DMR is part of a larger DMR that covers the *Gnasxl* promoter [14]). Paternal inheritance of the *Nespas^tm1Jop^* allele (hereafter called *ΔNespas*), a deletion of the *Nespas* DMR, results in loss of imprinting of *Nesp* and *Gnas*, and down regulation of *Gnasxl* [13]. The tissue specific imprinted expression of the *Gnas* transcript is controlled by a second differentially methylated region, the *Exon 1A* DMR that lies just upstream of *Gnas* exon 1 [15]. Paternal inheritance of the *Gnas^tm1Jop^* allele (hereafter called *ΔEx1A*), a deletion of the *Exon 1A* DMR, results in loss of imprinted expression of *Gnas* and so the *Exon 1A* DMR solely regulates the imprinted expression of *Gnas* [6], [16]. Thus the *Nespas* DMR must interact with the *Exon 1A* DMR to control the imprinted expression of *Gnas* although the mechanism of interaction is not yet clear.

PatDp(dist2) mice have two expressed copies of *Gnasxl* but lack expression of *Gnas* in imprinted tissues and also lack expression of *Nesp* [6], [13], [17] whereas MatDp(dist2) mice have two expressed copies of *Gnas* in imprinted tissues and two expressed copies of *Nesp* but lack expression of *Gnasxl*. PatDp(dist2) mice have greatly diminished expression of *Gnas* in newborn brown fat consistent with imprinted expression [6] and elevated levels of *Gnasxl* consistent with two expressed doses [13].

From studies of targeted deletions and an ENU induced mutation it is evident that both XLαs and Gsα play important roles in growth, behaviour and survival shortly after birth, but NESP55 does not [7], [9], [10], [18], [19]. Paternal inheritance of a null mutation in *Gnasxl* results in newborns with severely reduced suckling ability that become inert on the day of birth and most die within within a few days of birth [10]. Thus loss of expression of *Gnasxl* can account for much of the phenotype observed in MatDp(dist2) mice. On the other hand, maternal transmission of a null mutation resulting in loss of *Gnas* transcript gives rise to neonates with oedema and square shaped bodies, most of which die before weaning [7], [9] and maternal inheritance of a loss of function allele, *Gnas^Oedsml-mat^*, (*Oed*), results in gross neonatal oedema and pre-weaning lethality [18]. PatDp(dist2) are oedematous at birth [1], [20] (but the oedema is of considerably less severity than in *Oed* mice) [18] and oedema may account for at least part of the original description of a square shaped body [1], [21]. Thus the phenotype of mutants affecting Gsα function or expression has some similarity to that seen in PatDp(dist2). But, neither the null nor the loss of function mutation results in the hyperactivity shortly after birth that is probably the most distinctive feature of PatDp(dist2). So loss of maternal expression of *Gnas* transcripts in imprinted tissues can account for the oedematous phenotype seen in PatDp(dist2) mice but cannot account for the hyperactivity. Thus hyperactivity may be due to a double dose of *Gnasxl* alone or to the combined effect of a double dose of *Gnasxl* and loss of *Gnas* in imprinted tissues.

We have used genetic approaches to show the combination of over expression of *Gnasxl* and loss of *Gnas* gives rise to an early hyperactivity lethal phenotype. Furthermore when the expressed dosage of *Gnas* and *Gnasxl* is balanced then PatDp(dist2) are neither lethal nor hyperactive. Thus the dosage of *Gnas* and *Gnasxl* needs to be balanced for long term survival and normal activity. Furthermore PatDp(dist2) with balanced dosage but two doses of both *Gnasxl* and *Gnas* have normal viability and activity but preweaning growth retardation followed by catch up growth. Thus it may be important to have a single dose of *Gnasxl* and *Gnas* for normal development or there may be other genes involved in determining the PatDp(dist2) phenotype.

## Materials and Methods

### Ethics Statement

All mouse studies were carried out in accordance with the guidance issued by the Medical Research Council in “Responsibility in the use of animals in bioscience research (May 2008)”, were approved by the MRC Harwell ethical review committee and carried out under the authority of the UK Home Office Project Licence numbers 30/1518, 30/2065 30/2526, and 30/2642.

### Mice

Paternal duplication (PatDp(dist2)) mice were generated by the standard genetic method of intercrossing genetically marked translocation heterozygotes and identifying PatDp(dist2) mice with the aid of markers [22]. From these intercrosses up to 16% of live births are expected to be PatDp(dist2) [23]. We used the translocation T(2;8)26H, (T26H), which has a breakpoint on chromosome 2 at the *a*, nonagouti locus [23] proximal to the *Gnas* cluster.

To generate PatDp(dist2) with altered dosing of Gsα and XLαs we developed translocation stocks that were heterozygous for *ΔEx1A* (T26H +/+ *ΔEx1A*) (maternal allele listed first), homozygous for *ΔEx1A* (T26H *ΔEx1A*/+ *ΔEx1A*), heterozygous for *ΔNespas* (T26H +/+ *ΔNespas*), and heterozygous for both *ΔEx1A* and *ΔNespas* (T26H+*ΔEx1A*/+ *ΔNespas* +). PatDp(dist2) that were either heterozygous for *ΔEx1A*, or homozygous for *ΔEx1A*, or heterozygous for *ΔNespas*, or heterozygous for both *ΔEx1A* and *ΔNespas* were generated by crossing T26H +/++females with either TH26+/+ *ΔEx1A*, or T26H *ΔEx1A*/+ *ΔEx1A* or T26H +/+ *ΔNespas* or T26H+*ΔEx1A*/+ *ΔNespas*+males. For each cross, reciprocal crosses were also set up to generate PatDp(dist2) with wild-type alleles at *ΔEx1A* and/or *ΔNespas*. The genetic background of PatDp(dist2) mice was mixed but with major contributions from C3H/HeH (circa 45%) and the Harwell LL stock (circa 45%). Toe or tail clips were taken from offspring at birth and these served as both biopsies for identification and for genotyping. Offspring were genotyped for paternal duplication, *ΔEx1A* and *ΔNespas* as previously described [6], [13], [22].

### Phenotyping

Mice were classified for oedema, chunky appearance, activity, tail kink, and paddle feet, a paddling motion of the front feet, by daily visual observation from birth. Mice were removed from the box and observed for 2–3 minutes.

### RNA Analysis

Total RNA was extracted and northern analysis carried out as described previously (6, 13). Riboprobes specific for the unique first exons of *Gnas* and *Gnasxl* and for *Actb* have been previously described (6, 13). Transcript levels were measured by phosphoimager analysis and quantified as described previously (6,13).

### Biochemical Analysis of Plasma

Truncal blood from mice up to one week of age was collected using heparinised capillary tubes. Plasma was separated following centrifugation at 750×g for 3 minutes and stored at −80°C. Noradrenaline was measured in duplicate using an ELISA kit (Labor Diagnostika Nord GmbH & Co.KG) according to the manufacturer’s instructions. Blood glucose was measured using an Alphatrak Veterinary Blood Glucose Monitoring Meter Kit (Abbott) according to the manufacturer’s instructions. Insulin and glucagon were measured using an ELISA kit (Mercodia) according to the manufacturer’s instructions.

### Weight and Body Mass Index

Mice were weighed weekly from birth and from 4 weeks the length from the tip of the nose to the base of the tail was measured weekly. Body mass index was calculated using the following equation: weight (g)/(length (cm))^2^.

### Metabolic Caging

At nine weeks the mice were individually housed in metabolic cages for 24 h, during which time they had free access to a known amount of food and water. After 24 h, the amount of food and water consumed was measured along with the volume of urine produced. After housing in metabolic cages the mice were returned to their home cage. For a fuller protocol, see EMPRess (the European Phenotyping Resource for Standardised Screens from EUMORPHIA; http://empress.har.mrc.ac.uk).

### Metabolic Rate Measurements

At 10 and 21 weeks of age the mice were investigated using indirect calorimetry (Oxymax, Columbus Instruments, Columbus, OH, USA). Mice were weighed and then individually housed in the equipment for 24 h, with food and free access to water. After testing, the mice were returned to their home cage. Indirect calorimetry enabled measurement of oxygen consumption, carbon dioxide production, respiratory exchange ratio and heat production.

### DEXA Analysis

At 17 weeks of age the mice were weighed and given a recoverable anaesthetic prior to scanning using a General Electric Medical Systems Lunar PIXImus II X-ray densitometer (Inside Outside Sales, Fichburg, WI, USA), which allows the fat and lean mass and bone density to be calculated. Mice were placed in a heated box to aid recovery; once fully recovered, they were returned to their home cage.

### Statistical Analyses

Fisher’s exact test was used for comparisons of the incidence of PatDps and the occurrence of phenotypic features. Student’s *t* test (two-tailed) was used for assessing the results of the growth and metabolism studies and expression levels on northern blots. P values <0.05 were considered significant.

## Results

### Incidence and Survival of PatDp(dist2)

PatDp(dist2) has two expressed doses of *Gnasxl* and severely reduced expression of imprinted *Gnas* [6], [13] and will hereafter be referred to as PatDp(dist2)2∶0. To produce PatDp(dist2) with differing doses of *Gnasxl* and *Gnas* we utilised two mutants, *ΔEx1A* and *ΔNespas*. *ΔEx1A* is a deletion of the *Exon 1A* DMR, the region that regulates the imprinted expression of *Gnas*. On paternal inheritance *Gnas* is completely derepressed on the paternal allele [6]. Heterozygotes +/*ΔEx1A* (maternal allele listed first) show normal survival to birth for they were generated at a frequency of 44.5% (of 231 neonates) which is not significantly different from the expected 50% (P>0.05).The *ΔNespas* mutant allele is a deletion of the promoter and first exon of *Nespas*, the ICR for the *Gnas* cluster [13]. On paternal inheritance of *ΔNespas Gnasxl* is downregulated to about 24% of wild type levels (Figure S1) and *Gnas* is expressed to about 70% of wild type on the paternal allele. Heterozygotes +/*ΔNespas* also show normal survival to birth [13].

**Figure 2:**
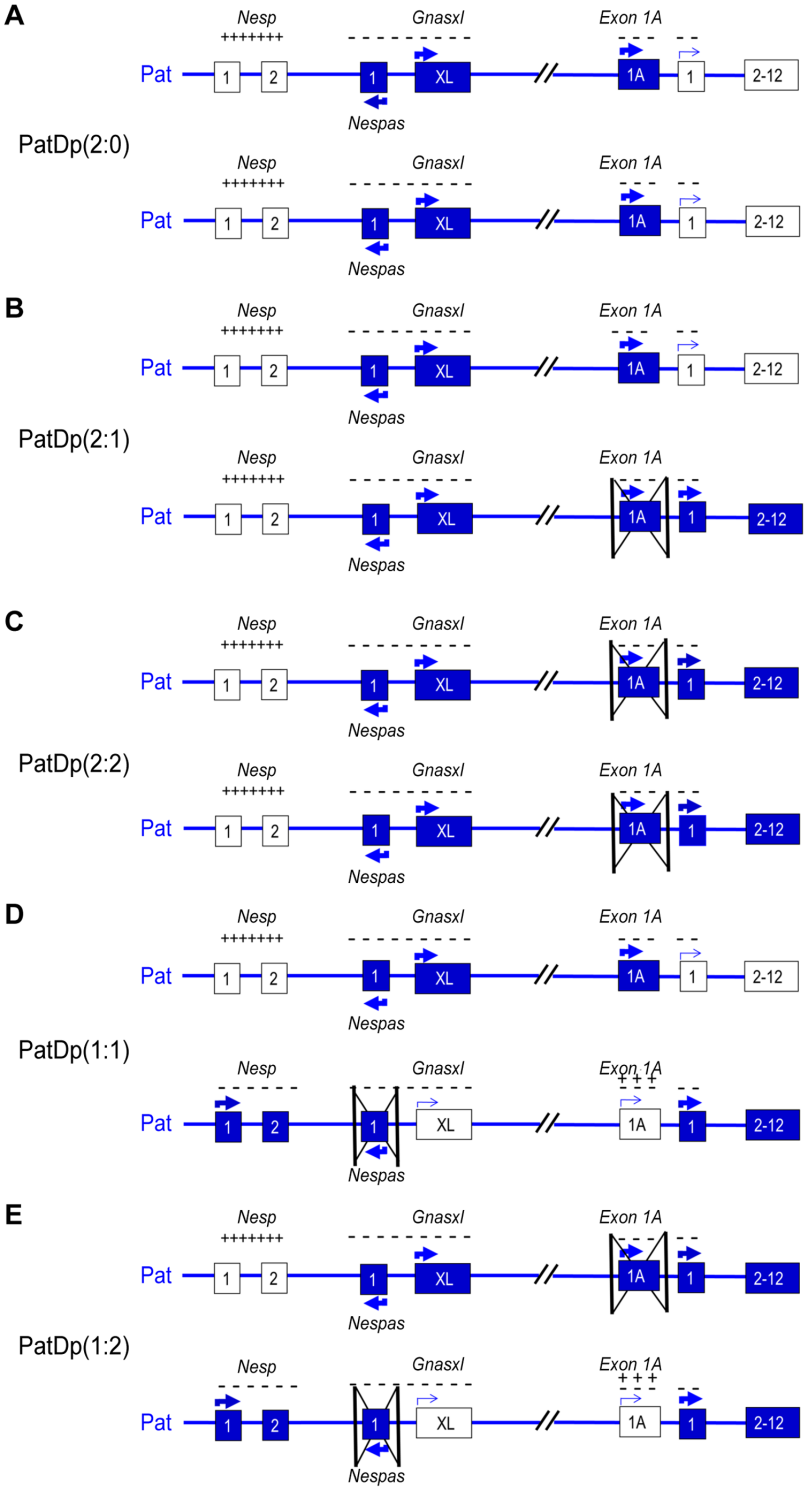
Transcript expression in PatDp(dist2). Summary of transcription and methylation at the *Gnas* cluster in (A) PatDp(dist2)2∶0, (B) PatDp(dist2)2∶1, (C) PatDp(dist2)2∶2 (D) PatDp(dist2)1∶1, (E) PatDp(dist2)1∶2. Transcribed exons are shown as filled boxes and nontranscribed exons or exons giving rise to very markedly reduced levels of transcript as empty boxes. *Nesp* exon 1 is noncoding and *Nespas* and *Exon 1A* give rise to noncoding transcripts. The arrows indicate the direction of transcription and a thin arrow indicates low expression. The methylation status of the *Exon 1A* DMR and the *Nespas* DMR is shown as plus signs for methylated and minus signs for unmethylated.

Four crosses were set up to generate PatDp(dist2) mice with altered dosing of *Gnasxl* and *Gnas* (Figure 2, Table 1). In Cross 1 PatDp(dist2) mice heterozygous for *ΔEx1A* were produced to provide PatDp(dist2)2∶1 with two predicted expressed doses of *Gnasxl* and one expressed dose of *Gnas*. In Cross 2 PatDp(dist2) mice homozygous for *ΔEx1A* were generated to produce PatDp(dist2)2∶2 with two predicted expressed doses of *Gnasxl* and two doses of *Gnas*. Cross 3 gave rise to PatDp(dist2) mice heterozygous for *ΔNespas* predicted to have slightly more than one expressed dose of *Gnasxl* and slightly less than one expressed dose of *Gnas*, called PatDp(dist2)1∶1. From Cross 4 PatDp(dist2) arose that were compound heterozygotes for *ΔEx1A* and *ΔNespas* predicted to have slightly more than one expressed dose of *Gnasxl* and slightly less than two expressed doses of *Gnas*, called PatDp(dist2)1∶2. For each cross reciprocal crosses were also set up resulting in PatDp(dist2)2∶0.

**Table 1:** Crosses to generate PatDp(dist2).

Cross	Cross	PatDp	Genotype of PatDp	Expected Ratio
				Gnasxl: Gnas
Control cross	T26H++/+++ X T26H++/+++	PatDp(dist2)2∶0	T26H++/+++	2∶0
Cross 1	T26H++/+++ X T26H++/++*ΔEx1A*	PatDp(dist2)2∶1	T26H++/++*ΔEx1A*	2∶1
Cross 2	T26H++/+++ X T26H +*ΔEx1A*/++*ΔEx1A*	PatDp(dist2)2∶2	T26H+*ΔEx1A*/++*ΔEx1A*	2∶2
Cross 3	T26H++/+++ X T26H++/+ *ΔNespas* +	PatDp(dist2)1∶1	T26H++/+ *ΔNespas* +	1∶1
Cross 4	T26H++/+++ X T26H+*ΔEx1A*/+ *ΔNespas* +	PatDp(dist2)1∶2	T26H +*ΔEx1A*/+ *ΔNespas* +	1∶2

**Figure 3:**
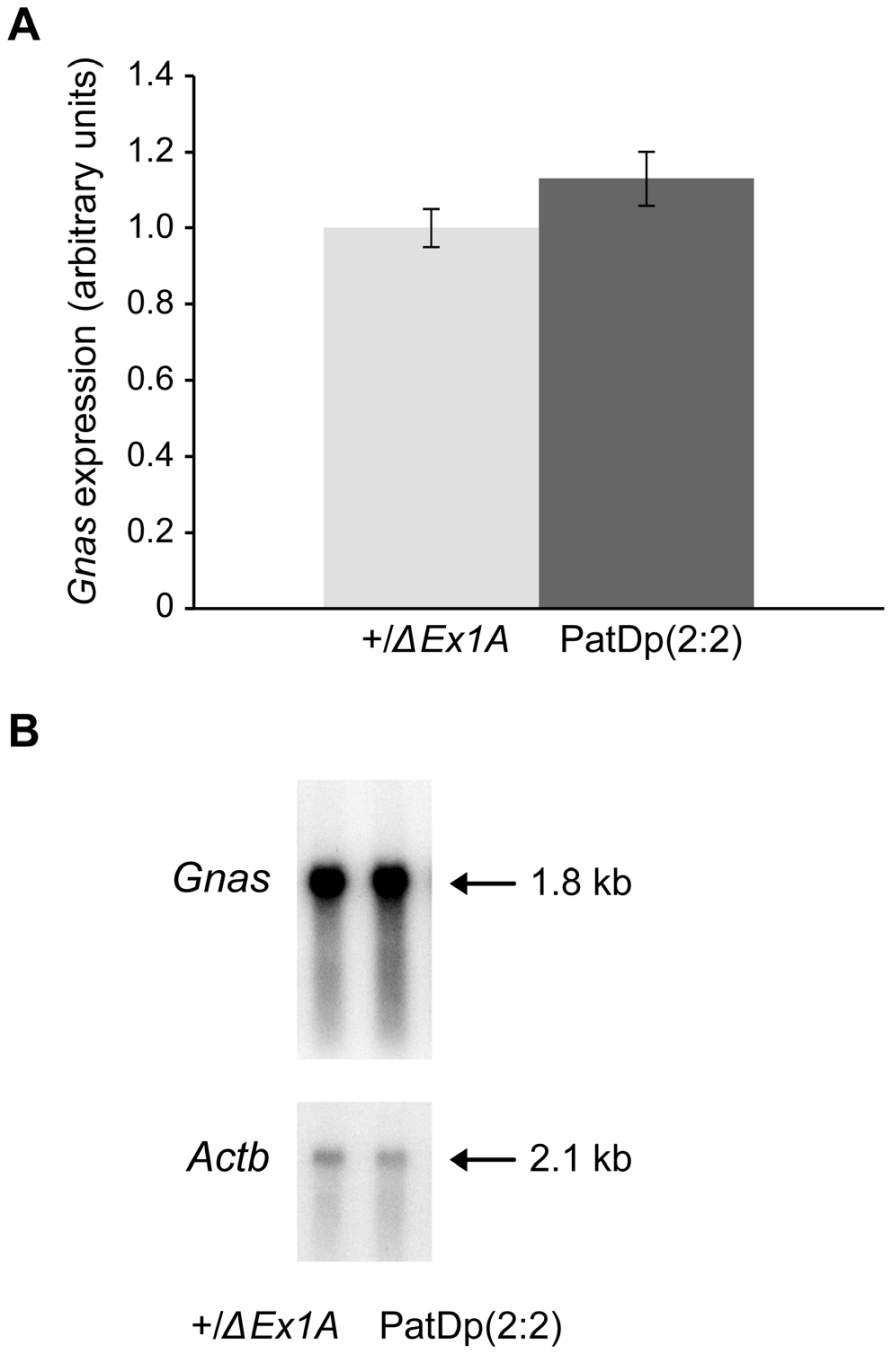
*Gnas* expression in newborn brown fat measured by northern blotting. (**A**) Bar chart showing *Gnas* levels in PatDp(2∶2) normalised to *β-actin* mRNA and shown relative to the level in +/*ΔEx1A*. Mean ± sem calculated for 5 PatDp(dist2)2∶2 and 8+/*ΔEx1A*. (**B**) Northern blot of *Gnas* and *β-actin* loading control in +/*ΔEx1A* and PatDp(2∶2) using 5 µg total RNA from newborn brown fat.

Heterozygotes +/*ΔEx1A* were known to express two doses of imprinted *Gnas*, and PatDp(dist2)2∶1 and PatDp(dist2)1∶1 to express one dose of imprinted *Gnas* [6]. From northern analysis, similar levels of *Gnas* were found in newborn brown fat from +/*ΔEx1A* and PatDp(dist2)2∶2 homozygous for *ΔEx1A* indicating that PatDp(dist2)2∶2 express two full doses of *Gnas* (Figure 3).

There were no significant differences in the incidence or phenotypes of PatDp(dist2)2∶0 from the reciprocal crosses and so the data from these were pooled for the Control cross (Table 1, Figure 2A **)**. The incidence of PatDp(dist2)2∶0 generated using the T26H translocation was not significantly different from that found previously [24]. There was no significant difference in the incidence of PatDp(dist2)2∶2 compared to PatDp(dist2)2∶0, nor in the incidence of PatDp(dist2)2∶1 or PatDp(dist2)1∶2, but the incidence of PatDp(dist2)1∶1 at birth was significantly greater than PatDp(dist2)2∶0 (P = 0.0394, (Fisher’s exact test, two tailed) (Table 2).

**Table 2:** Incidence and Survival of PatDp(dist2).

Cross	PatDp	Expected Ratio	Incidence at birth		Survival to 7d		Survival to weaning	
		Gnasxl: Gnas	No	%	No	%	No	%
Control cross	PatDp(dist2)2∶0	2∶0	31/428	7	3/28	8	0/28	0
Cross 1	PatDp(dist2)2∶1	2∶1	*18/171	10	5/14	36	0/14	0
Cross 2	PatDp(dist2)2∶2	2∶2	19/171	11	^,^ 412/12	100	^3^^,^ 511/12	92
Cross 3	PatDp(dist2)1∶1	1∶1	∼ ^1^31/258	12	^3^^,^ 419/21	90	^3^^,^ 519/21	90
Cross 4	PatDp(dist2)1∶2	1∶2	∧6/73	8	^2^ 5/6	83	^3^^,^ 4 5/6	83

* 23 PatDp altogether but 2 were PatDp(dist2)2∶2 and 3 were PatDp(dist2)2∶0.

∼ 36 PatDp altogether but 3 were PatDp(dist2)0∶2 and 2 were PatDp(dist2)2∶0.

∧ 10 PatDp altogether but 3 were PatDp(dist2)2∶1 and one was not classified for *ΔEx1A.*

Incidence No shows the number of PatDps born/total number of mice born.

Survival No shows number of PatDps/total number of PatDps scored.

For comparison of PatDp(dist2)2∶0 with other PatDp classes:

1 P<0.05,

2 P<0.01,

3 P<0.0001 (Fisher’s exact test, 2 tailed).

For comparison of PatDp(dist2)2∶1 with other PatDp classes except PatDp(dist2)2∶0:

4 P<0.002,

5 P<0.0001 (Fisher’s exact test, 2 tailed).

It should be borne in mind that the incidence of PatDp(dist2)2∶1, PatDp(dist2)1∶1 and PatDp(dist2)1∶2 is likely to be an underestimate because of recombination between the TH26 breakpoint on chromosome 2 and the *Gnas* cluster which lie 21cM apart (www.informatics.jax.org) (May, 2012). Thus in Cross 1, the cross to generate PatDp(dist2)2∶1, recombinants PatDp(dist2)2∶0 and PatDp(dist2) 2∶2 were found. In Cross 3, the cross to generate PatDp(dist2)1∶1, recombinants PatDp(dist2)2∶0 and PatDp(dist2)0∶2 occurred. In Cross 4, the cross to generate PatDp(dist2)1∶2, recombinants PatDp(dist2)2∶2 arose. All recombinants in Cross 2, the cross to generate PatDp(dist2)2∶2, will be identical and indistinguishable from non recombinant PatDp(dist2)2∶2 (Table 2). In the absence of recombination 4 more PatDp(2∶1) were expected in Cross 1, 6 more PatDp(dist2)1∶1 in Cross 3 and 1 more PatDp(dist2)1∶2 in Cross 4. Using the expected numbers of PatDps that would have occurred in the absence of recombination the incidence of PatDp(dist2)2∶1 is also significantly greater than PatDp(dist2)2∶0 (P = 0.0259, (Fisher’s exact test, two tailed)). Overall the results indicate that dosage of *Gnasxl* and *Gnas* can affect survival of PatDp(dist2) to birth.

Interestingly a recombinant PatDp(dist2)0∶2 homozygous for *ΔNespas* that arose in Cross 3 had a normal phenotype and survived for 6 months. This is probably because in such a mouse the expected levels of *Gnasxl* expression would be nearly 50% of the wild type level and this appears to be sufficient for viability.

In agreement with earlier work PatDp(dist2)2∶0 generated using the T26H translocation only survived for a few days [24]. However there was some increase in survival of PatDp(dist2)2∶1 compared to PatDp(dist2)2∶0 with a couple of PatDp(dist2)2∶1 reaching 13 days but none survived to weaning (Table 2). For PatDp(dist2)2∶2 and PatDp(dist2)1∶1 where the expressed doses of *Gnas* and *Gnasxl* are expected to be in balance and in PatDp(dist2)1∶2 predicted to have one expressed dose of *Gnasxl* and two expressed doses of *Gnas*, there was a remarkable increase in survival with most PatDp(dist2)2∶2, PatDp(dist2)1∶1 and PatDp(dist2)1∶2 reaching weaning (Table 2). After weaning the viability of many of these PatDp(dist2) was as good as their non PatDp(dist2) littermates. Thus for long term survival relative overexpression of *Gnasxl* is disadvantageous but overexpression of imprinted *Gnas* is not.

### Neonatal Phenotype

At birth the PatDp(dist2)2∶0 mice had the typical phenotype (Table 3). Thus, most were noted to be oedematous although oedema is much less marked in PatDp(dist2)2∶0 than in the *Oed* mutant, a point mutation in *Gnas* exon 6 [18], [25]. Most PatDp(dist2)2∶0 were described as chunky and over 90% were observed to be hyperactive within 24 hours of birth. The hyperactivity is very distinctive. Whereas non PatDp mice move very little shortly after birth, PatDp(dist2)2∶0 tend to be in motion most of the time, circling or crawling, often rapidly, with the forelimbs showing a paddling motion (paddle feet). They frequently fall on their backs and thrash around but are able to right themselves. The hyperactive behaviour becomes more extreme with the passing days (Video S1, S2). About half were noted to have a tail kink and paddle feet. The occurrence of each of these features is highly significant (P<0.0001, Fisher’s exact test, two tailed) for none of these features was observed in the 351 non duplication mice generated in the cross.

**Table 3:** Neonatal Phenotype of PatDp(dist2).

PatDp	Expected Ratio	Oedema		Chunky		Hyperactive				Tail kink		Paddle feet	
	Gnasxl: Gnas	No	%	No	%	day 1		day 2		No	%	No	%
						No	%	No	%				
PatDp(dist2)2∶0	2∶0	22/25	88	20/24	83	14/21	67	15/16	93	12/20	60	11/20	55
PatDp(dist2)2∶1	2∶1	*0/14	0	∼5/14	36	∼2/13	15	*3/8	37	6/14	43	4/14	29
PatDp(dist2)2∶2	2∶2	*0/19	0	* ^2^0/19	0	*0/17	0	*1/12	8	* ^3^1/19	5	* ^3^ 0/19	0
PatDp(dist2)1∶1	1∶1	*0/28	0	* ^2^1/28	18	*0/28	0	*1/20	5	* ^3^2/28	7	*2/28	7
PatDp(dist2)1∶2	1∶2	*0/6	0	∼1/6	17	∼0/6	0	*0/6	0	∧0/6	0	∧0/6	0

For comparison of PatDp(dist2)2∶0 with other PatDp classes:

* P<0.001;

∼ P<0.01,

∧ P<0.05 (Fisher’s exact test, 2 tailed).

For comparison of PatDp(dist2)2∶1 with other PatDp classes except PatDp(dist2)2∶0:

^2^P<0.01;

^3^P<0.05 (Fisher’s exact test, 2 tailed).

No indicates number of PatDp with the phenotype/total number of PatDp classified.

Of note, none of the PatDp mice with either one or two doses of *Gnas*, thus PatDp(dist2)2∶1, PatDp(dist2)2∶2, PatDp(dist2)1∶1 or PatDp(dist2)1∶2 were oedematous (Table 3). As oedema is known to be associated with loss of functional imprinted *Gnas* in the presence of a single expressed dose of *Gnasxl* [7], [9], [25], [26] it can be concluded that the oedema seen in PatDp(dist2)2∶0 is attributable to lack of expression of *Gnas* in tissues in which it is imprinted and not to overexpression of *Gnasxl*.

Even though oedema was absent in PatDp(dist2)2∶1, a number were noted to be chunky (Table 3), indicating that oedema and a chunky appearance are probably two separate components of the short square body shape phenotype seen in PatDp(dist2)2∶0 [1].

There was also a diminution in the number of PatDp(dist2)2∶1, PatDp(dist2)2∶2, PatDp(dist2)1∶1 and PatDp(dist2)1∶2 described as chunky compared to PatDp(dist2)2∶0. In addition, fewer PatDp(dist2)2∶2 and PatDp(dist2)1∶1 were chunky compared to PatDp(dist2)2∶1 (Table 3). Overall these results indicate that a double dose of expressed *Gnasxl* together with loss of expression of imprinted *Gnas* gives rise to this phenotype. However, if the double dose of *Gnasxl* is matched with a double dose of *Gnas* the chunky phenotype was not seen. For normality the expressed doses of *Gnasxl* and *Gnas* need to be balanced (Table 3).

Hyperactivity in PatDp(dist2)2∶0 although manifested on the day of birth, becomes fully developed by the next day (Table 3). There was a decrease in the incidence of hyperactivity in PatDp(dist2)2∶1, PatDp(dist2)2∶2, PatDp(dist2)1∶1 and PatDp(dist2)1∶2 both on the day of birth and the following day. By day 2 almost all PatDp(dist2)2∶0 were hyperactive but hyperactivity was virtually absent in PatDp(dist2)2∶2, PatDp(dist2)1∶1 and PatDp(dist2)1∶2. A single PatDp(dist2)1∶1 was found to be hyperactive on day two, but appeared normal by day four and this mouse was also classified as chunky. These findings were unexpected as *Gnas* and *Gnasxl* have the same predicted expressed dosage in PatDp(dist2)1∶1 as in wild type. Loss of functional imprinted *Gnas* in the presence of a single expressed dose of *Gnasxl* is not associated with hyperactivity in the first few days of life [7], [18] although tremor and ataxia starting at around one week of age have been observed in one *Gnas* knockout model [7]. On the other hand hyperactivity in the first few days has been observed in another mutant which like PatDp(dist2)2∶0 has a double dose of *Gnasxl* and loss of *Gnas* in imprinted tissues [27]. Overall the results indicate that overexpression of *Gnasxl* combined with loss of imprinted *Gnas* expression is responsible for the hyperactivity phenotype.

The incidences of tail kink and paddle feet, both known features of PatDp(dist2)2∶0, are not significantly different in PatDp(dist2)2∶1 but are reduced in PatDp(dist2)2∶2, PatDp(dist2)1∶1 and PatDp(dist2)1∶2. Furthermore, fewer PatDp(dist2)2∶2 and PatDp(dist2)1∶1 exhibited either tail kink and/or paddle feet than PatDp(dist2)2∶1 (Table 3). Overall these results indicate that a double dose of expressed *Gnasxl* together with either loss of, or a single dose of imprinted *Gnas* gives rise to this phenotype. For normality the expressed doses of *Gnasxl* and *Gnas* need to be balanced (Table 3).

Thus oedema can be attributed to lack of expression of *Gnas* in tissues in which it is imprinted, chunkiness, hyperactivity, tail kink and paddle feet to overexpression of *Gnasxl*, combined with loss of imprinted *Gnas* expression.

### Altered Metabolism in Neonatal PatDp(dist2)2∶0 but not PatDp(dist2)2∶2

**Figure 4:**
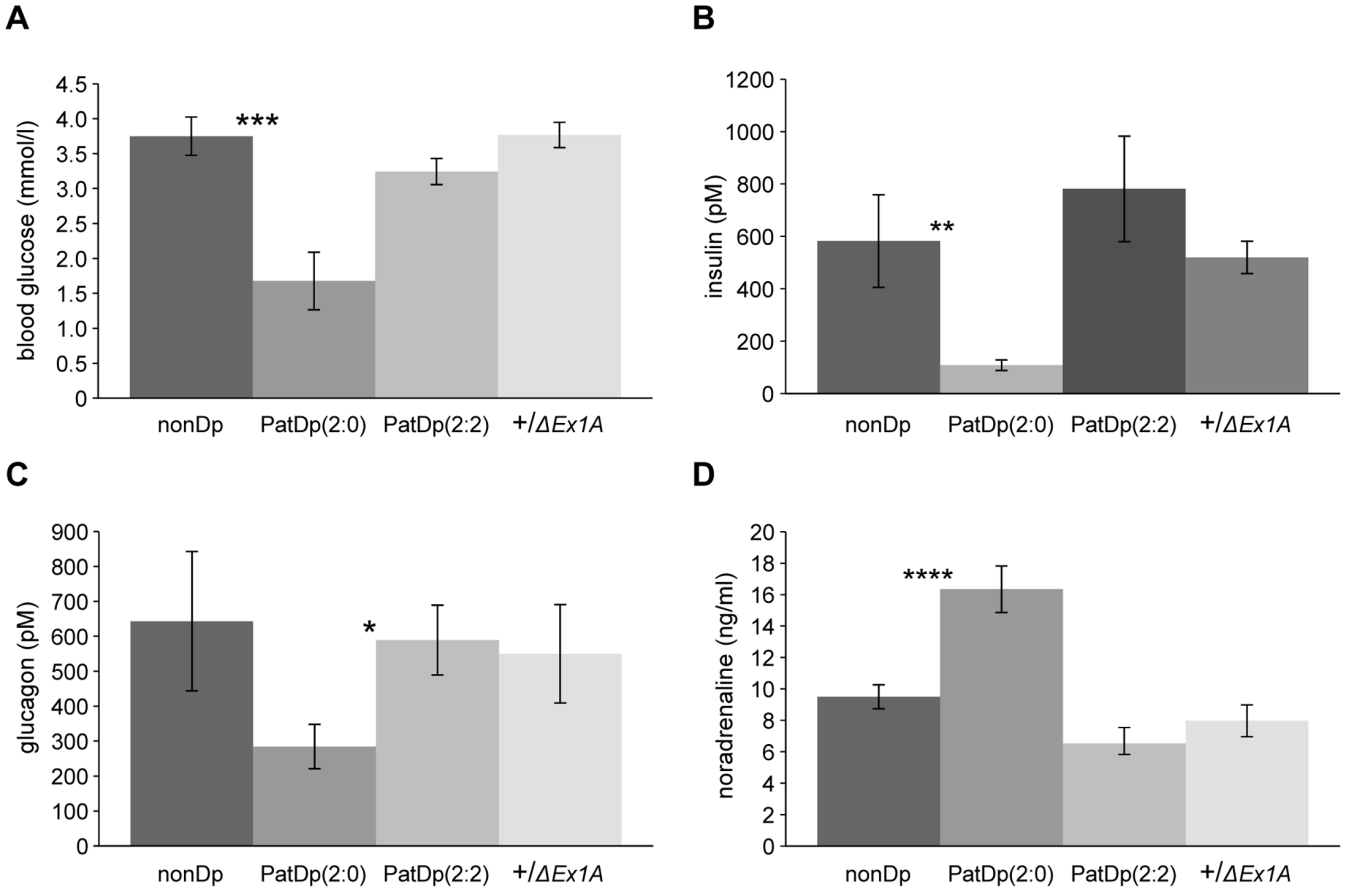
Metabolic analyses in early postnatal stages. (**A**) blood glucose levels. (n = 9–10) (**B**) plasma insulin levels.(n = 10–14) (**C**) plasma glucagon levels.(n = 9–13) (**D**) plasma noradrenaline. (n = 10–38). Error bars show standard errors of the means; ***P<0.001, **P<0.01, *P<0.05.

Studies of metabolism were undertaken in PatDp(dist2)2∶0 and and PatDp(dist2)2∶2 and their nonduplication litttermates. The nonduplication littermates of PatDp(dist2)2∶0 are wild type for *ΔEx1A* whereas nonduplication littermates of PatDp(dist2)2∶2 are heterozygous +/*ΔEx1A* and thus have biallelic *Gnas* expression. We tested neonates for glucose and key hormones involved in energy homeostasis (Figure 4). In PatDp(dist2)2∶0 blood glucose was considerably reduced and accordingly, plasma insulin was also low compared to their nonduplication sibs. Furthermore, the plasma glucagon level, expected to be raised in response to hypoglycaemia, was under half that found in nonduplication +/+ sibs. Plasma noradrenaline levels, expected to be raised in response to hypoglycaemia were elevated by more than 60% in PatDp(dist2)2∶0. In contrast PatDp(dist2)2∶2 had normal levels of glucose, insulin, glucagon and noradrenaline. Thus there is an overall disruption in glucose metabolism in PatDp(dist2)2∶0 attributable to overexpression of *Gnasxl* combined with loss of expression of *Gnas*. Loss of *Gnasxl* expression in *Gnasxl^m+/p−^* pups also results in hypoglycaemia but, surprisingly no elevation of plasma noradrenaline in newborns [10]. In the current study plasma noradrenaline was measured in 2 and 3 day old pups (P2 and P3) and no statistically significant difference was found in *Gnasxl^m+/p−^* and wild type littermates. The combined data for P2 & P3 were: 6.57 ng/ml ±0.67 versus 8.85±1.50; WT versus *Gnasxl^m+/p−^*; mean±s.e.m; n = 14 WT and 9 *Gnasxl^m+/p−^;* P = 0.19).

In newborn +/*ΔEx1A* the levels of glucose, insulin and glucagon and noradrenaline were not significantly different from wild type indicating that overexpression of *Gnas* is not associated with altered glucose metabolism.

### Both PatDp(dist2)2∶2 and +/*ΔEx1A* Mice Show Postnatal Growth Retardation

Further studies from birth to adulthood of PatDp(dist2)2∶2 and their nonduplication +/*ΔEx1A* sibs were carried out. In addition studies of +/*ΔEx1A* and their +/+ sibs from a cross of C3H/HeH females to +/*ΔEx1A* males were undertaken. For weight studies between birth and weaning the weights of both sexes were combined as no significant differences in the weights of males and females were found, and after weaning only males were used. Mice were weighed on the day of birth and weekly thereafter.

**Figure 5:**
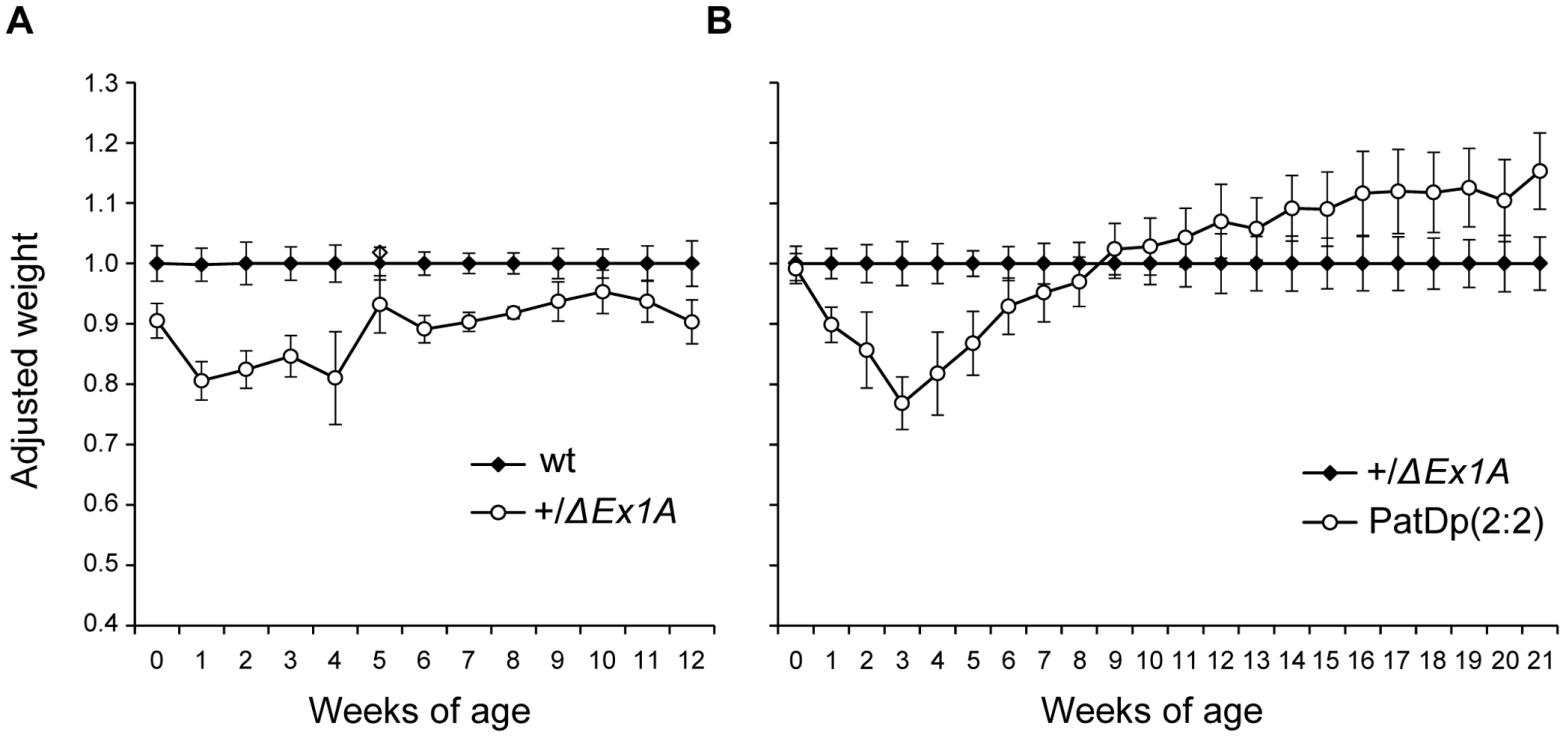
Growth retardation. (**A**) Growth curve of +/*ΔEx1A* and wild-type littermates from 1 day to 12 weeks. The weights of wild-type littermates have been normalised to 1 at each timepoint and the weights of +/*ΔEx1A* mice have been taken as a percentage of wild-type weights. (n = 5–26). (**B**) Growth curve of PatDp(dist2)2∶2 and +/*ΔEx1A*. The weights of +/*ΔEx1A* littermates have been normalised to 1 at each timepoint and the weights of PatDp(dist2)2∶2 mice have been taken as a percentage of +/*Ex1A* weights (n = 9–12). Error bars show standard errors of the means.

The weights are shown as a percentage of their wild type siblings (Figure 5). The +/*ΔEx1A* mice were lighter at birth, 90.1% (P = 0.021), of the weight of their wild type siblings, indicating prenatal growth retardation. Growth retardation became more pronounced during the first week of life but by weaning +/*ΔEx1A* had started to catch up so that by nine weeks their weight was no longer significantly different from wild type **(** Figure 5A **)**. Similar findings of prenatal and preweaning growth retardation followed by catch up growth has been reported on paternal inheritance of the *Ex1A-T-CON* allele which also results in loss of imprinted expression of *Gnas* [28].

PatDp(dist2)2∶2 were generated by Cross 2, T26H++/+++ X T26H+*ΔEx1A*/++*ΔEx1A* so all the nonduplication sibs were heterozygous for *ΔEx1A*. PatDp(dist2)2∶2 did not differ in weight from their nonduplication +/*ΔEx1A* sibs at birth or at one week of age indicating that PatDp(dist2)2∶2 were also growth retarded and to the same extent as +/*ΔEx1A*. By two weeks of age PatDp(dist2)2∶2 were even lighter in weight than +/*ΔEx1A* and continued to weigh less than +/*ΔEx1A* until 6 weeks but subsequently did not differ significantly in weight from +/*ΔEx1A*. Thus postnatal growth retardation in PatDp(dist2)2∶2 peaked at three weeks and was followed by catch up growth (Figure 5B).

Thus loss of imprinting of *Gnas* in +/*ΔEx1A* results in growth retardation before weaning resulting in proportionately smaller mice followed by catch up growth in adulthood. PatDp(dist2)2∶2 with predicted balanced expressed dosage of *Gnas* and *Gnasxl* show a more severe pre-adult growth retardation resulting in proportionately smaller mice followed by catch up growth by 10–11 weeks of age and overall increased weight thereafter.

The body length of +/*ΔEx1A* mice, their wild type sibs, and PatDp(dist2)2∶2 and their +/*ΔEx1A* sibs was measured weekly from 4 weeks. +/*ΔEx1A* mice were shorter (93.2±0.5% s.e.m of the length of wild type sibs) than their wild type sibs at 4 weeks (P<0.05, n = 4–11) but caught up in length by 10 weeks. PatDp(dist2)2∶2 were even shorter than +/*ΔEx1A* from 4 to 6 weeks (91.8±1.3% s.e.m at 4 weeks P = 0.00097, n = 10, and 96.8±1.2% s.e.m at 6 weeks P<0.022, n = 12) but thereafter did not differ in length from +/*ΔEx1A*.

Body mass index, BMI was significantly lower in +/*ΔEx1A* than wild type at 4 weeks (0.29±0.004 s.e.m versus 0.34±0.007 s.e.m; P = 2.55E-05, n = 4–11) but did not differ from wild type thereafter. No difference in BMI was found between +/*ΔEx1A* and PatDp(dist2)2∶2 (n = 10–12).

Analysis of 24h food intake at 10 weeks showed no difference in +/*ΔEx1A* and wild type sibs (n = 4–14) or in +/*ΔEx1A* and PatDp(dist2)2∶2 (n = 10–12) (data not shown).

The results of DEXA analysis at 17 weeks indicated there were no significant differences in fat or lean mass in +/*ΔEx1A* and wild type sibs (n = 5–12) or in +/*ΔEx1A* and PatDp(dist2)2∶2 (n = 9) (data not shown).

### +/*ΔEx1A* but not PatDp(dist2)2∶2 have a Higher Metabolic Rate

**Figure 6:**
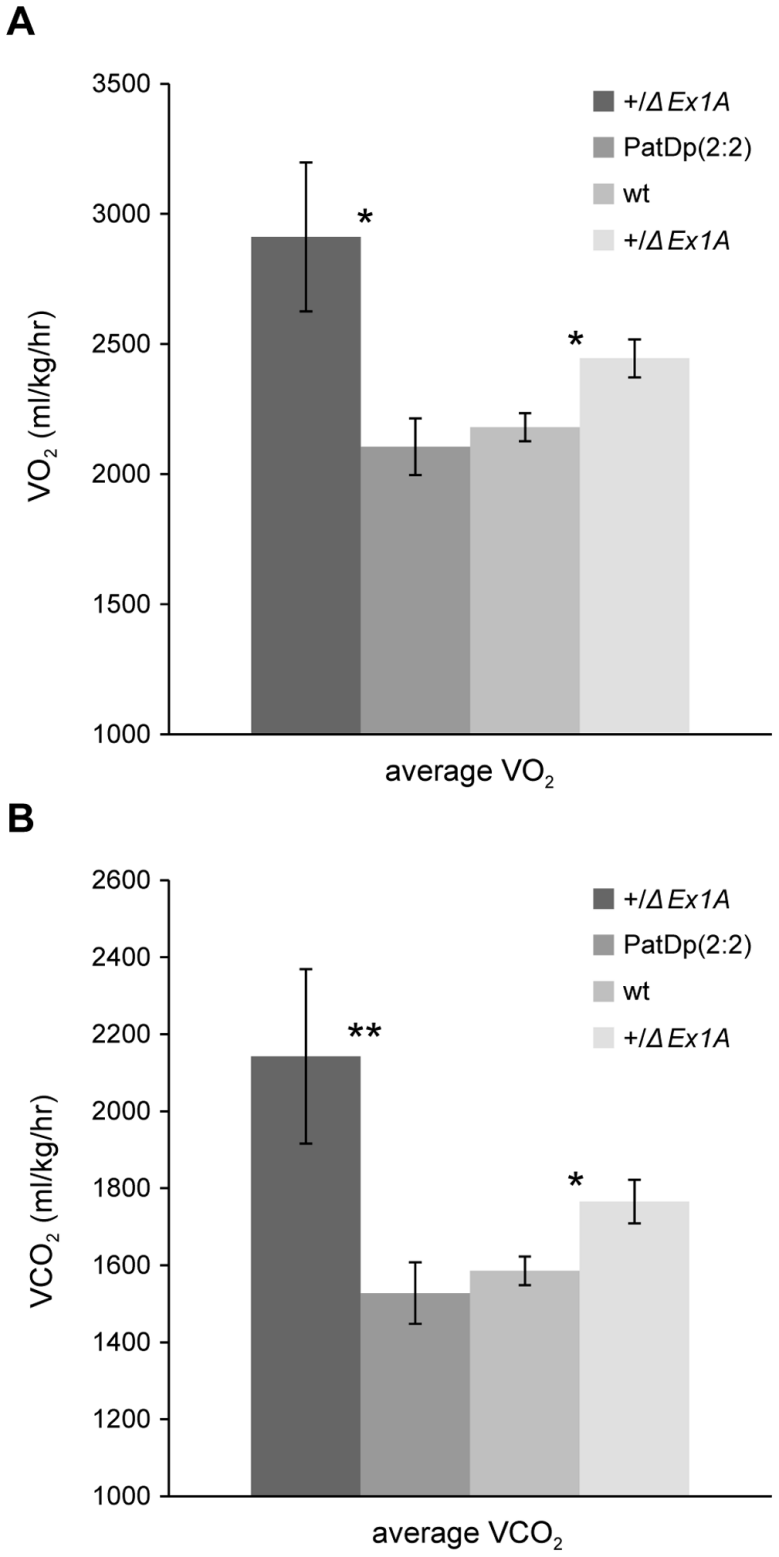
Metabolic rate. Analysis of metabolic rate in 21 week old male +/*ΔEx1A* and their wild-type littermates, and PatDp(dist2)2∶2 and their +/*ΔEx1A* littermates. (A) and (B) Rates of oxygen consumption and carbon dioxide output respectively (n = 4–12). Error bars show standard errors of the means; **P<0.01, *P<0.05.

At 10 weeks of age +/*ΔEx1A* tended to show increased oxygen consumption and carbon dioxide output in comparison with wild-type littermates and these increases were statistically significant (P<0.03) by 21 weeks of age in +/*ΔEx1A* mice indicative of a raised metabolic rate (Figure 6).

## Discussion

The oedema in PatDp(dist2)2∶0 is likely to be due to loss of expression of imprinted *Gnas* as oedema was not seen in PatDp(dist2) in which *Gnas* expression was restored to normal. This agrees with the results from other studies indicating that null or loss of function mutations of *Gnas* are associated with neonatal oedema [1], [7], [9], [26], [27], [29] but neonatal oedema was not observed when imprinted *Gnas* expression was restored to normal [6], [30]. Oedema in PatDp(dist2)2∶0 is known to develop from 12.5 dpc and by late gestation PatDp(dist2)2∶0 are severely oedematous and *Oed* even more so with oedema declining somewhat in both genetic conditions before birth [18], [20]. The occurrence of oedema *in utero* could conceivably affect survival to birth and so the restoration of imprinted *Gnas* expression in PatDp(dist2)2∶1 and PatDp(dist2)1∶1 may account for their increased incidence at birth compared to PatDp(dist2)2∶0.

The other neonatal phenotypes of PatDp(dist2), namely lethality, hyperactivity, paddle feet, chunky appearance, tail kink, can be attributed to biallelic expression of *Gnasxl* combined with loss of imprinted *Gnas* expression. A considerable improvement in the incidence of newborns that were neither hyperactive nor chunky and some improvement in survival to seven days was found when biallelic expression of *Gnasxl* was combined with normal monoallelic expression of *Gnas* in PatDp(dist2)2∶1. However, for full viability and loss of hyperactivity, biallelic expression of *Gnasxl* needs to be balanced with biallelic expression of imprinted *Gnas* or monoallelic expression of *Gnasxl* with monoallelic expression of *Gnas*. This need for balanced dosage is in accord with observations indicating that maternally expressed *Gnas* and paternally expressed *Gnasxl* act antagonistically [10].

Interestingly monoallelic expression of *Gnasxl* combined with biallelic, and thus overexpression of imprinted *Gnas*, in either PatDp(dist2)1∶2 or +/*ΔEx1A* results in normal viability. Normal viability was also recently reported for the *Ex1A-T-CON* mutant in which *Gnas* is overexpressed [28]. Thus overexpression of *Gnas* has less drastic results on phenotype than overexpression of *Gnasxl*. But in the presence of two expressed copies of *Gnasxl* it is critical to have two expressed copies of *Gnas* in order to survive to weaning.

PatDp(dist2)2∶0 are hyperactive and hypoglycaemic and both these phenotypes may contribute to their early death. Hyperactivity has also been reported for some *ΔNesp55^m^* neonates [27] which like PatDp(dist2)2∶0 have two expressed doses of *Gnasxl* and no expressed doses of imprinted *Gnas*. The hyperactivity in PatDp(dist2)2∶0 and *ΔNesp55^m^* may reflect a central neurological deficit. The finding of raised noradrenaline in PatDp(dist2)2∶0 together with a recent report showing that *Gnasxl* is expressed in neonatal muscle [31] may well have relevance for the hyperactivity phenotype. Conversely the hypotonia and inactivity of *Gnasxl* KO pups might be at least partly due to the lack of XLαs in muscle.

PatDp(dist2)2∶0 are hypoglycaemic with low insulin and raised noradrenaline. *ΔNesp55^m^* neonates are also hypoglycaemic and although poor feeding may well contribute to their hypoglycaemia they may, in addition, have a defect in glucose counterregulation [26]. The causes of the hypoglycaemia in PatDp(dist2)2∶0 are not known, and even though they have a milk spot they could have a feeding impairment that could contribute to the hypoglycaemia. Also, in view of their low glucagon, PatDp(dist2)2∶0 could have a defect in glucose counterregulation. In PatDp(dist2)2∶2 with balanced expressed doses of both *Gnasxl* and *Gnas*, glucose metabolism is restored to normal, suggesting that both *Gnasxl* and imprinted *Gnas* have a role in neonatal glucose metabolism.

Both Gsα and XLαs have several variants. Thus C-terminally truncated neural specific forms GsαN1 and XLN1 of Gsα and XLαs respectively are known [32], [33]. In addition XLαs has a second ORF that encodes a protein ALEX and then there is an N-terminally extended form of XLαs called XXLαs [34], [35], [36]. On comparison of the phenotypes seen in mouse models in which different transcripts have been inactivated it appears that XLαs/XXLαs play a part in growth and metabolism, ALEX may affect suckling but roles for XLN1 and GsαN1 have not been established yet [28], [37]. Possibly the metabolic defects in PatDp(dist2)2∶0 can be attributed in part at least to overexpression of XLαs/XXLαs but the contribution of the various *Gnasxl* proteins to other aspects of the PatDp(dist2)2∶0 phenotype is as yet unknown.

Our data indicate that both *Gnas* and *Gnasxl* have roles in the phenotype of PatDp(dist2). Loss of the orthologous *GNAS* transcript in humans is associated with a number of endocrine and bone disorders including pseudohypoparathyroidism, Albright Hereditary Ostedystrophy and progressive osseous heteroplasia [38]. Pseudohypoparathyroidism type Ia (PHP-Ia) is due to maternal inheritance of an inactivating mutation in *GNAS* and pseudohypoparathyroidism type Ib is caused by defective imprinting at the cluster (reviewed in [37]). In both conditions there is resistance to parathyroid hormone but PHP-Ia is also associated with multiple hormone resistance and Albright hereditary osteodystrophy (AHO). Some patients with PHP-Ib have mild AHO features indicating overlap in the PHP-Ia and PHP-Ib phenotypes [39], [40], [41], [42], [43]. Pseudohypoparathyroidism type Ib, PHP-Ib, can occur sporadically or as an autosomal dominant. One cause of the sporadic form is paternal uniparental disomy for human chromosome 20 (patUPD20) and altogether there are reports of eight patUPD20 patients with PHP-Ib [40], [44], [45], [46], [47]. The molecular consequences for patUPD20 appear to the similar to those for PatDp(2∶0) and are predicted to result in overexpression of *GNASXL* and loss of expression of *GNAS* in imprinted tissues. Autosomal dominant PHP-Ib is caused by maternal inheritance of microdeletions with microdeletions within the *GNAS* cluster resulting in epigenetic changes leading to loss of imprinted *GNAS* expression together with overexpression of *GNASXL* or microdeletions upstream of the cluster resulting in loss of imprinted *GNAS* expression alone [48], [49], [50], [51]. Thus the clinical features of PHP-Ib appear to be due to loss of imprinted *GNAS*, and overexpression of *GNASXL* does not appear to play a significant role in the phenotype. However two of the patUPD20 patients showed neonatal hypoglycaemia [40], [45] which, in view of the findings in PatDp(dist2)2∶0 in the mouse, could be due to overexpression of *GNASXL*. In neonatal PatDp(dist2) mice hyperactivity commencing shortly after birth and elevated noradrenaline are also features of raised *Gnasxl* levels. It would be of interest to ascertain if metabolism and activity in PHP-Ib patients are affected in the early postnatal period and if so, whether elevated *GNASXL* is responsible.

We have shown that loss of imprinting of *Gnas* in +/*ΔEx1A* results in postnatal growth retardation followed by catch up growth and can be attributed solely to loss of imprinting, resulting in biallelic expression, of *Gnas*. The effects on postnatal growth in +/*ΔEx1A* parallel the growth pattern seen in +/*Ex1A-T-CON* mice which also have loss of imprinting of *Gnas* [28]. Thus maternally expressed *Gnas* clearly has a role in growth. Paternally expressed *Gnasxl* is also known to affect postnatal growth and mutations resulting in loss of functional *Gnasxl* also result in postnatal growth retardation [7], [10], [11], [18], [28], [29], [52] The finding that both overexpression of *Gnas* and underexpression of *Gnasxl* results in growth retardation accords with the parental conflict theory that predicts that maternally expressed imprinted genes will be growth inhibiting and paternally expressed imprinted genes will be growth enhancing [12]. Thus it was expected that postnatal growth would be unaffected in PatDp(dist2)2∶2 mice which have a predicted balanced dosage of *Gnas* and *Gnasxl*. However PatDp(dist2)2∶2 mice show a more extreme postnatal growth retardation than seen with overexpression of *Gnas* in +/*ΔEx1A*. Thus biallelic expression of both *Gnas* and *Gnasxl* has resulted in postnatal growth retardation. One interpretation is that it is important to have monoallelic expression of antagonistic *Gnasxl* and *Gnas* for normal growth. The advantages of monoallelic expression of both *Gnasxl* and *Gnas* must outweigh the costs associated with functional haploidy.

## Supporting Information

Figure S1 **Expression of** ***Gnasxl*** **was reduced on paternal inheritance of** ***ΔNespas***. (**A**) Northern blot of *Gnasxl* and *β-actin* loading control using 2.5 µg of poly (A)^+^ RNA from 15.5-dpc embryos. MatDp(dist2) have no expressed copies of *Gnasxl,* whilst PatDp(dist2) have two expressed copies, leading to the absence of the 2.5 kb band and the presence of a strong 2.5 kb band respectively. (**B**) Bar chart showing the *Gnasxl* expression levels in +/*ΔNespas* (mean ± s.e.m 24±3.56%) were decreased on comparison to wild-type (+/+) (mean ± s.e.m 103±9.39%) *P = 0.005 (Student’s *t* test, two-tailed). The mean ± s.e.m was calculated for 3 wild-type (+/+) and four +/*ΔNespas*.
(TIF)

Video S1 **Hyperactivity in PatDp(dist2).** Video of a 6 day old PatDp(dist2) and wild-type littermate showing almost continual activity and rapid movement with paddling of the front feet in the PatDp(dist2) but very little movement in the wild-type. The PatDp(dist2) has the characteristic tail kink or bend, and by 6 days is clearly smaller than the wild-type but oedema and a chunky appearance are no longer evident. The mice have a genetic background that is 50% *Mus musculus castaneus* and 50% laboratory mouse. On this background there was slightly better survival of PatDp(dist2) to 7 days (10/23) compared with 3/28 on an entirely laboratory mouse background (P = 0.0106 (Fisher’s exact test, 2-tailed) but no difference in survival to weaning.
(MP4)

Video S2 **Hyperactivity in PatDp(dist2).** Video of the same PatDp(dist2) and wild-type littermate at 6 days as in Video S2 and the PatDp(dist2) at 5 days of age on its own. The video shows the ability of the PatDp(dist2) to right itself after falling on its back, as well as its continual activity and rapid movement.
(MP4)
