# Supplementary figures and images for: Gene Dosage Effects at the Imprinted Gnas Cluster
Source: PLoS One. 2013 Jun 18;8(6):e65639. doi: 10.1371/journal.pone.0065639 (PMC3688811; doi:10.1371/journal.pone.0065639)

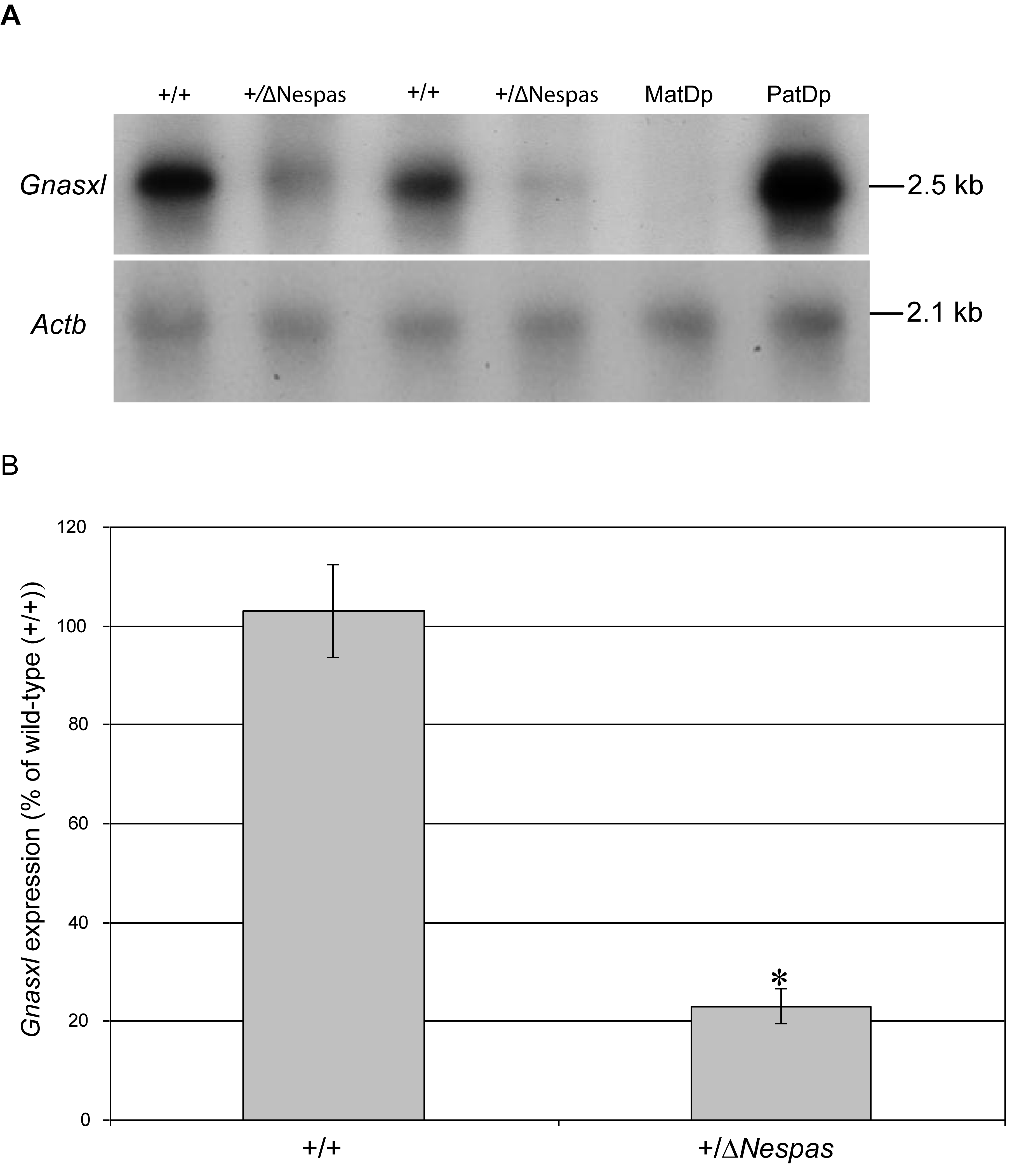

Supplement: Figure S1 — Expression of Gnasxl was reduced on paternal inheritance of ΔNespas. (A) Northern blot of Gnasxl and β-actin loading control using 2.5 µg of poly (A)+ RNA from 15.5-dpc embryos. MatDp(dist2) have no expressed copies of Gnasxl, whilst PatDp(dist2) have two expressed copies, leading to the absence of the 2.5 kb band and the presence of a strong 2.5 kb band respectively. (B) Bar chart showing the Gnasxl expression levels in +/ΔNespas (mean ± s.e.m 24±3.56%) were decreased on comparison to wild-type (+/+) (mean ± s.e.m 103±9.39%) *P = 0.005 (Student’s t test, two-tailed). The mean ± s.e.m was calculated for 3 wild-type (+/+) and four +/ΔNespas. (TIF) [file pone.0065639.s001.tif]
